# Targeted and untargeted metabolomics uncovering the effects of Jiawei Ermiao Granule on patients with persistent HPV infection

**DOI:** 10.3389/fcimb.2025.1550908

**Published:** 2025-04-07

**Authors:** Xiu Li, Li Ning, Hongting Zhao, Yating Xu, Yu Si, Jiayi Hua, Qingling Ren

**Affiliations:** ^1^ Jiangsu Clinical Medicine Innovation Center for Obstetrics and Reproduction, Affiliated Hospital of Nanjing University of Chinese Medicine, Nanjing, Jiangsu, China; ^2^ The First Clinical Medical School, Nanjing University of Chinese Medicine, Nanjing, Jiangsu, China; ^3^ Department of Gynecology, Affiliated Hospital of Nanjing University of Chinese Medicine, Nanjing, Jiangsu, China

**Keywords:** Jiawei Ermiao Granule, HPV, untargeted metabolomics, targeted metabolomics, amino acid metabolism, tyrosine

## Abstract

Jiawei Ermiao Granule (JWEMG) is a traditional Chinese herbal formulation widely used in China for the treatment of human papilloma virus (HPV) infections. However, the mechanisms underlying their efficacy in clearing HPV infections remain unclear. This study aimed to elucidate the mechanisms by which JWEMG clears persistent HPV infections from a metabolomics perspective using modern analytical techniques. Untargeted and targeted metabolomics analyses were performed on vaginal lavage samples from 33 patients using liquid chromatograph mass spectrometer (LC-MS) and hydrophilic interaction liquid chromatography-mass spectrometry (HILIC-MS). Untargeted metabolomics identified 47 potential biomarkers through volcano plot analysis, among which 30 exhibited a reversal trend following JWEMG intervention. Kyoto Encyclopedia of Genes and Genomes (KEGG) pathway analysis suggested that JWEMG may exert therapeutic effects on patients with persistent HPV infections via pathways related to starch and sucrose metabolism, galactose metabolism, fructose and mannose metabolism, and amino sugar and nucleotide sugar metabolism. Targeted metabolomics revealed a significant increase in tyrosine levels in the vaginal/cervical microenvironment following JWEMG treatment. By integrating targeted and untargeted metabolomics, this study provides a comprehensive exploration of the holistic effects of JWEMG on HPV-infected patients, addressing the challenges of scientifically explaining the pharmacological mechanisms of multi-component, multi-target traditional Chinese medicines.

## 1 Introduction

HPV infection is a common lower genital tract infection in women. In 1972, virologist Harald zur Hausen detected and isolated HPV in cervical cancer tissues, establishing that cervical cancer is primarily caused by HPV infection (zur Hausen, 2002). Most HPV infections are asymptomatic and self-resolve within six months to one year due to rapid immune clearance (Loopik et al., 2021). However, individuals with persistent high-risk HPV (HR-HPV) infections may progress to cervical cancer if timely screening and interventions are not implemented (Jain et al., 2023). Currently, there are no specific antiviral drugs for HPV infections (Perkins et al., 2020). Existing therapeutic options can be broadly categorized into four groups: immune enhancers, antimetabolites, *Lactobacillus* preparations, and novel biological agents. However, most of these are topical treatments, which, if misused, may cause local mucosal damage, impair the immune function of the mucosal barrier, and even exacerbate the condition. Moreover, these therapies are often associated with limitations such as prolonged treatment regimens and suboptimal efficacy. Surgical interventions also face challenges, including the presence of residual lesions and recurrence post-operation. The recurrence rate of cervical lesions following conization has been reported to range from 7% to 25% (Fan et al., 2018). Thus, identifying safe and effective therapeutic strategies is an urgent priority.

Metabolomics is an emerging systems biology approach that analyzes endogenous small-molecule metabolites and their associated metabolic pathways, offering valuable insights into the relationships between metabolites and physiological or pathological changes, as well as drug mechanisms of action (Wawrzyniak et al., 2024). It facilitates tracking disease progression, devising rational management plans, identifying drug targets for new drug development, and discovering biomarkers for early prediction, diagnosis, and classification of diseases (Steffens et al., 2010). As an integral part of precision medicine, metabolomics provides crucial technical support for research. Metabolomics comprises untargeted and targeted approaches. Untargeted metabolomics involves comprehensive and systematic analysis of small-molecule metabolites in cells, plasma, tissues, or entire organisms. Targeted metabolomics focuses on high-sensitivity, high-precision analysis of specific classes of metabolites, typically those with similar physicochemical properties or involvement in related metabolic pathways (e.g., amino acids, carbohydrates, and lipids) (Pité et al., 2018). Combining both approaches allows for a more comprehensive and in-depth understanding of the mechanisms of traditional Chinese medicines, thereby supporting their modernization and application in clinical practice.

Traditional Chinese medicine (TCM) has emerged as a promising therapeutic approach for addressing the complex pathological changes associated with HPV infection (Lin et al., 2017). JWEMG, an empirically derived prescription for HPV infection, is based on the classical formula Er Miao San for treating damp-heat conditions. JWEMG is composed of eight Chinese herbal medicines: *Phellodendron chinense* Schneid., *Atractylodes lancea* (Thunb.) DC., *Atractylodes macrocephala* Koidz., *Paris polyphylla* Smith var. *yunnanensis* (Franch.) Hand.-Mazz., *Oldenlandia diffusa* (Willd.) Roxb., *Isatis indigotica* Fort., *Smilax glabra* Roxb., and *Coix lacryma-jobi* L. var. *ma-yuen* (Roman.) Stapf (Pharmacopoeia of the People’s Republic of China, 2020). As a hospital formulation, JWEMG has been utilized in clinical settings for the treatment of HPV infections for several decades, with demonstrated efficacy. In a study of 97 HPV-infected patients, JWEMG achieved a viral clearance rate of 76.67% after 6-12 months of treatment (Hong, 2017). Similarly, in 80 patients with persistent HR-HPV infections, the overall effective rate reached 82.5% (Shen, 2022). Furthermore, among 60 patients with persistent HR-HPV positivity after loop electrosurgical excision procedure (LEEP) surgery, the clearance rate was 82.76% following JWEMG treatment (Chen, 2020).

Consequently, this study utilized mass spectrometry to perform targeted and untargeted metabolomic analyses on vaginal lavage samples collected before and after treatment with JWEMG in patients with persistent HPV infection. The aim was to identify the potential mechanisms of action of JWEMG, providing data to support drug efficacy evaluation, elucidation of disease mechanisms, and the development of individualized treatment strategies.

## 2 Materials and methods

### 2.1 Drugs and reagents

JWEMG (Batch number: Z20210019000) was provided by the Jiangsu Provincial Hospital of Traditional Chinese Medicine, affiliated with Nanjing University of Chinese Medicine. The total weight of a single prescription was 100 g, and the proportions of the eight medicinal ingredients in JWEMG are shown in **Table 1**. The high-performance liquid chromatography (HPLC) fingerprint profile of JWEMG was established in our team’s previous research (Zou et al., 2018).

**Table 1:** Composition of JWEMG.

Chinese name	Full name	Medicinal part	Proportion (%)
Huangbo	*Phellodendron chinense* Schneid.	Phellodendri Chinensis Cortex	10%
Cangzhu	*Atractylodes lancea* (Thunb.) DC.	Atractylodis Rhizoma	10%
Baizhu	*Atractylodes macrocephala* Koidz.	Atractylodis Macrocephalae Rhizoma	10%
Chonglou	*Paris polyphylla* Smith var. *yunnanensis* (Franch.) Hand.-Mazz.	Paridis Rhizoma	10%
Baihuasheshecao	*Oldenlandia diffusa* (Willd.) Roxb.	Whole plant	10%
Banlangen	*Isatis indigotica* Fort.	Isatidis Radix	10%
Tufuling	*Smilax glabra* Roxb.	Smilacis Glabrae Rhizoma	10%
Yiyiren	*Coix lacryma-jobi* L.var.*ma-yuen* (Roman.) Stapf	Coicis Semen	30%

Acetonitrile (ACN), methanol, and methyl tert-butyl ether (MTBE) were purchased from Merck (Darmstadt, Germany). BSTFA containing 1% trimethylchlorosilane (TMCS), and methoxyamine hydrochloride were obtained from Sigma-Aldrich (MO, USA).

Amino acid standards, including alanine (3.08 mg/mL), valine (3.10 mg/mL), isoleucine (1.94 mg/mL), glycine (3.47 mg/mL), aspartic acid (3.15 mg/mL), glutamic acid (1.49 mg/mL), tyrosine (1.11 mg/mL), proline (3.23 mg/mL), phenylalanine (1 mg/mL), lysine (1 mg/mL), asparagine (0.95 mg/mL), histidine (2.97 mg/mL), serine (3.56 mg/mL), leucine (3.96 mg/mL), cystine (2.01 mg/mL), and L-tyrosine (1.50 mg/mL), were all purchased from Sigma-Aldrich (MO, USA).

### 2.2 Statistical power analysis and sample size calculation

A statistical power analysis was performed to determine the appropriate sample size for this study. Based on our previous research (Wu Jieping Medical Foundation, Ministry of Science and Technology, Grant No. 350.6750.15015), the clearance rates of HR-HPV after six months of treatment with Baofukang Suppository and JWEMG were 38.57% (*p*_0_ = 0.3857) and 85.29% (*p*_1_ = 0.8529), respectively. Using a significance level (α) of 0.05, a power (1-β) of 80%, and accounting for a 20% dropout rate, the calculated minimum sample size was approximately 20 participants per group. In this study, we enrolled 40 participants, which exceeds the minimum sample size required to ensure adequate statistical power.

### 2.3 Study population and design

**Figure 1:**
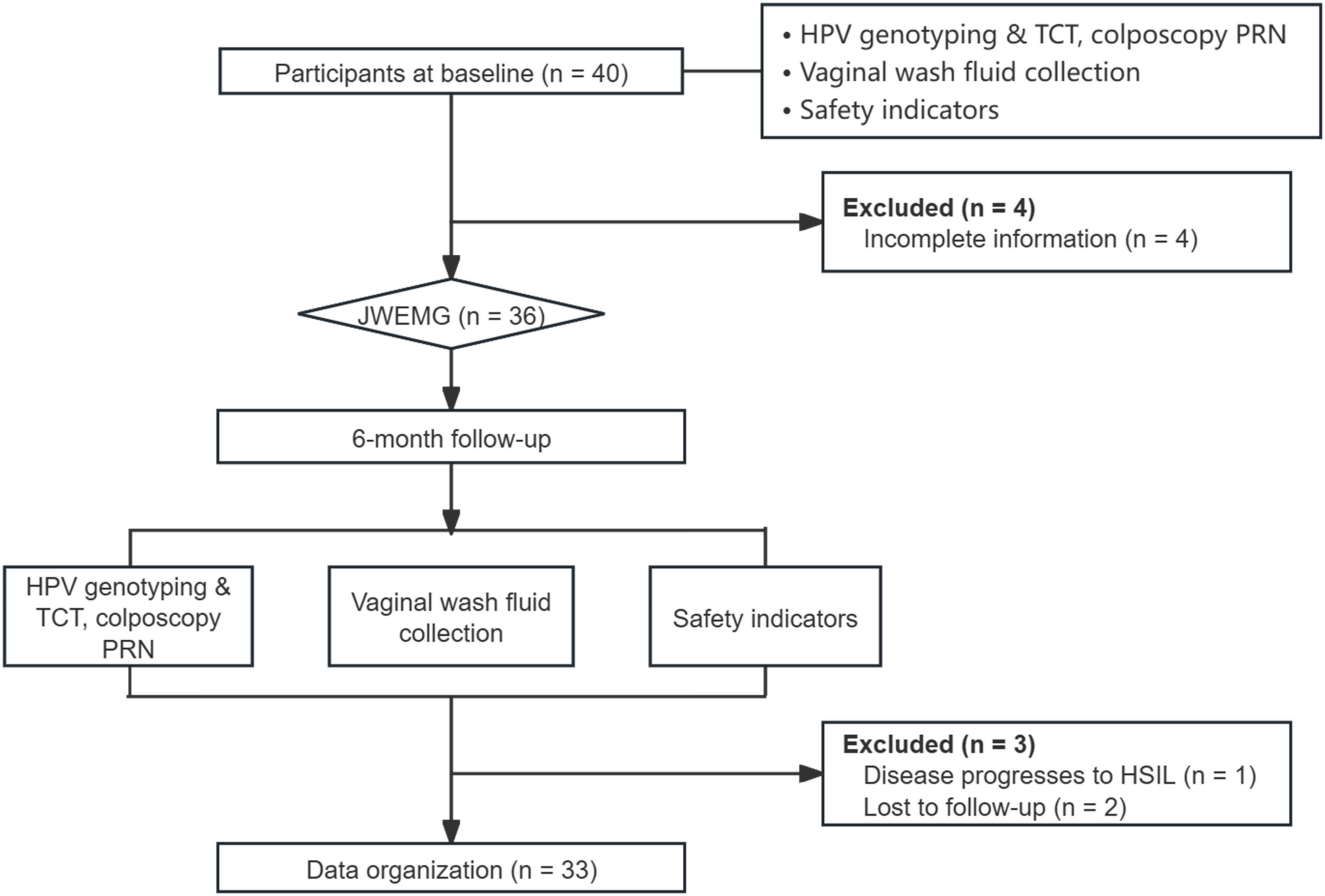
Clinical research flowchart.

Forty patients attending the Department of Gynecology at Jiangsu Provincial Hospital of TCM between December 2022 and December 2023 were enrolled in this study. Eligibility criteria were as follows: (1) age 30–65 years with a minimum of one year of regular sexual activity; (2) consecutive HR-HPV positivity confirmed by two HPV tests (≥ 6 months apart) with cytological or histopathological diagnosis of low-grade squamous intraepithelial lesion (LSIL) or lower; (3) diagnosis of damp-heat syndrome based on traditional Chinese medicine criteria, characterized by symptoms such as increased vaginal discharge, yellow discoloration, and itching; (4) provision of informed consent and willingness to participate. Exclusion criteria comprised: (1) any cervical treatment within the past year; (2) history of sexually transmitted infections, including Chlamydia, Gonorrhea, Syphilis, Genital herpes, Trichomoniasis, or other STIs; (3) immune system disorders or immunosuppressant use; (4) severe comorbidities interfering with treatment; (5) known allergy to Chinese herbal medicine; (6) psychiatric disorders or inability to cooperate. All participants voluntarily joined and provided written informed consent. The study received Ethics Committee approval from Jiangsu Provincial Hospital of TCM (Ethics Approval No.: 2021NL-025-03) and adhered to the principles of the Helsinki Declaration. Patients diagnosed with HPV infection were treated with JWEMG, which was self-administered orally twice daily. Each treatment cycle spanned three months, with two cycles administered consecutively (paused during menstruation) before re-assessment. On enrollment and at six months post-JWEMG initiation, samples of vaginal lavage fluid, and venous blood were collected. HPV and Thinprep cytologic test (TCT) tests were performed concurrently. A detailed clinical trial flowchart is provided in **Figure 1**.

### 2.4 Baseline data collection and specimen collection

Baseline data, including demographic characteristics, reproductive history, and contraceptive methods, were gathered using structured questionnaires. Specimen collection was conducted after confirming patients were not menstruating, pregnant, postpartum, or using vaginal irrigation or medication within the past 48 hours and had abstained from sexual activity for at least three days. Patients were positioned in the lithotomy position to fully expose the vaginal side and posterior fornix. Following this, the vaginal wall and cervix were irrigated with 7 mL of 0.9% sodium chloride solution, with 5–6 mL of lavage fluid withdrawn and preserved at -80°C. Cervical surface secretions were cleared, and cervical samples were obtained using a cervical brush, with subsequent cytological examination conducted by three senior pathologists at Jiangsu Provincial Hospital of TCM. Cytological diagnostics followed the TBS classification guidelines from the International Cancer Society. Concurrently, HPV detection and TCT were performed on specimens preserved in separate cell preservation media. The HPV detection method used is approved by both the Food and Drug Administration (FDA) and the China National Medical Products Administration.

Additionally, venous blood samples were collected from the antecubital vein for further analysis. Safety assessments were conducted by the laboratory department of Jiangsu Provincial Hospital of TCM.

### 2.5 Sample preparation

#### 2.5.1 Sample preparation for untargeted metabolomics

500 μL of vaginal lavage fluid was dried to a powder. 20 μL of LC-MS grade water was added to re-dissolve the sample, followed by vortexing for 5 minutes. To each sample, 225 μL of methanol was added, mixed, and vortexed for 3 minutes, then 750 μL of MTBE was added, vortexed for 10 seconds, and incubated at 4°C for 10 minutes. 188 μL of HILIC-MS grade water was then added, vortexed for 20 seconds, and the mixture was centrifuged at 18,000 rpm at 4°C for 2 minutes. 150 μL of the layer was transferred for drying. The dried sample was dissolved in 110 μL of acetonitrile-water (8:2, v/v), vortexed for 10 minutes, and sonicated for 10 minutes. The mixture was then centrifuged at 13,000 rpm for 10 minutes, and the supernatant was injected into LC-MS for analysis.

#### 2.5.2 Sample preparation for targeted metabolomics

After thawing the collected vaginal lavage fluid, 500 μL was transferred into an EP tube and stored at -80°C. The sample in the EP tube was placed in a lyophilizer overnight until it dried to a powder. Then, 50 μL of LC-MS grade water was added to re-dissolve the sample, followed by vortexing for 5 minutes. To each sample, 200 μL of methanol containing an internal standard was added, vortexed for 3 minutes, and centrifuged at 18,000 rpm for 10 minutes at 4°C. 100 μL of the supernatant was transferred to a new EP tube and dried using a lyophilizer. After drying, 30 μL of methoxyamine (10 mg/mL) was added, and the mixture was shaken at 30°C for 1.5 hours, followed by the addition of 30 μL of BSTFA, and shaking at 37°C for 0.5 hours. Finally, the mixture was centrifuged at 18,000 rpm for 10 minutes, and the supernatant was collected for HILIC-MS analysis.

### 2.6 LC-MS and HILIC-MS analysis

#### 2.6.1 LC-MS analysis

The analysis was performed using a Waters Acquity UPLC BEH C18 column (2.1 × 100 mm, 1.7 μm). The mobile phase A consisted of 0.1% formic acid in water, and mobile phase B was a mixture of 0.1% formic acid and 100% acetonitrile. For positive ion mode, both A and B contained 10 mM ammonium formate and 0.1% formic acid. For negative ion mode, both A and B contained 10 mM ammonium acetate. The flow rate was maintained at 0.35 mL/min, with a column temperature of 45°C and an autosampler temperature of 4°C. The gradient elution program was as follows: 0–1 min, 95% A, 5% B; 1–5 min, 80% A, 20% B; 5–12 min, 60% A, 40% B; 12–18 min, 40% A, 60% B; 18–20 min, 20% A, 80% B; 20–22 min, 95% A, 5% B.

#### 2.6.2 HILIC-MS analysis

The analysis was performed using an Atlantis Silica HILIC column (2.1 mm × 150 mm, 5.0 μm; packing material: unmodified high-strength silica particles). The mobile phase A consisted of water-acetonitrile (95:5, v/v) with 10 mmol/L ammonium acetate, and phase B consisted of acetonitrile-water (95:5, v/v) with 10 mmol/L ammonium acetate. The gradient elution program was as follows: 0–6 min, 98% B to 60% B; 6–8 min, 60% B; 8–8.1 min, 60% B to 98% B; 8.1–10 min, 98% B. The flow rate was 0.25 mL/min, and the column temperature was maintained at 60°C.

### 2.7 Data analysis

Molecular features of a wide range of metabolites were extracted, followed by visual validation of the extracted results. To minimize systematic variations across samples, the SERRF normalization method was applied, combined with quality control (QC) samples to eliminate systematic errors in the raw data. The processed data matrix was then imported into MetaboAnalyst (https://www.metaboanalyst.ca/) for univariate statistical analysis, including principal component analysis (PCA) and orthogonal partial least squares discriminant analysis (OPLS-DA). Differential variables identified were validated between the control and model groups using *t*-test, with *p* < 0.05 and Fold change ≥ 1.2 or ≤ 0.8333 considered as potential biomarkers. Identified potential biomarkers were further subjected to Gene Ontology (GO) enrichment and KEGG pathway analyses in MetaboAnalyst to identify related metabolic pathways. For targeted metabolomics analysis, data were organized using Excel and imported into MetaboAnalyst for clustering analysis, with *p* < 0.05 considered indicative of significant biomarkers.

### 2.8 Statistical analysis

Data analysis was conducted using GraphPad Prism 9.00, with values expressed as mean ± SD. For multiple group comparisons, one-way analysis of variance (ANOVA) was used if the data were normally distributed with homogeneity of variance. Multiple comparisons were performed using multifactorial ANOVA, and pairwise comparisons were conducted using one-way ANOVA. Non-parametric tests were used for data that did not meet normality or homogeneity requirements. A *p*-value < 0.05 was considered statistically significant.

## 3 Results

### 3.1 HPV clearance rate following treatment with JWEMG

Firstly, we summarized the demographic and clinical information of 33 patients, including age, BMI, parity, disease duration, postcoital bleeding, etc. As shown in **Table 2**, the average age among HR-HPV-infected patients was 44.76 ± 10.94 years, with a BMI of 22.73 ± 1.91. Our findings demonstrated that after six months of treatment with oral JWEMG, the HPV clearance rate reached 75.76%. Other safety indicators, such as alanine aminotransferase (ALT), aspartate transaminase (AST), uric acid (UA), and serum creatinine (Scr), remained within normal ranges and showed no statistically significant changes.

**Table 2:** Demographic and clinical indicators of patients prior and post treatment.

Basic information			Prior treatment	Post treatment	*p*-value
Age (years), mean ± SD		44.76 ± 10.94	/	/	/
BMI (kg/m^2^), mean ± SD		22.73 ± 1.91	/	/	/
Gravidity, mean ± SD		3.00 ± 1.48	/	/	/
Parity, mean ± SD		1.30 ± 0.64	/	/	/
Disease duration (months), mean ± SD		16.27 ± 4.12	/	/	/
Condom use ^&^, n (%)		6 (18.18%)	/	/	/
Postcoital bleeding ^#^, n (%)		3 (9.09%)	/	/	/
HPV16 or HPV18 infection, n (%)		4 (12.12%)	/	/	/
Patterns of HPV infection, n (%)	Single infection	19 (57.58%)	/	/	/
	Dual infection	12 (36.36%)	/	/	/
	Multiple infection	2 (6.06%)	/	/	/
Efficacy of patients with HPV infection					
Complete viral clearance, n (%)		/	/	19 (57.58%)	/
Partial viral clearance, n (%)		/	/	6 (18.18%)	/
Persistent infection, n (%)		/	/	8 (24.24%)	/
Overall efficacy, n (%)		/	/	25 (75.76%)	/
Safety indicators					
ALT (U/L), mean ± SD		/	21.26 ± 7.01	20.71 ± 6.04	0.6302
AST (U/L), mean ± SD		/	22.02 ± 5.83	21.77 ± 5.47	0.8043
UA (μmol/L), mean ± SD		/	234.00 ± 52.75	224.99 ± 51.39	0.3255
Scr (μmol/L), mean ± SD		/	69.55 ± 14.37	70.86 ± 13.39	0.5924

^&^Condom use refers to consistent use during the study period.

^#^Postcoital bleeding refers to at least one occurrence during the study period.

### 3.2 Metabolic pathway alterations in persistent HPV infection patients treated with JWEMG using untargeted metabolomics

#### 3.2.1 Method validation

**Figure 2:**
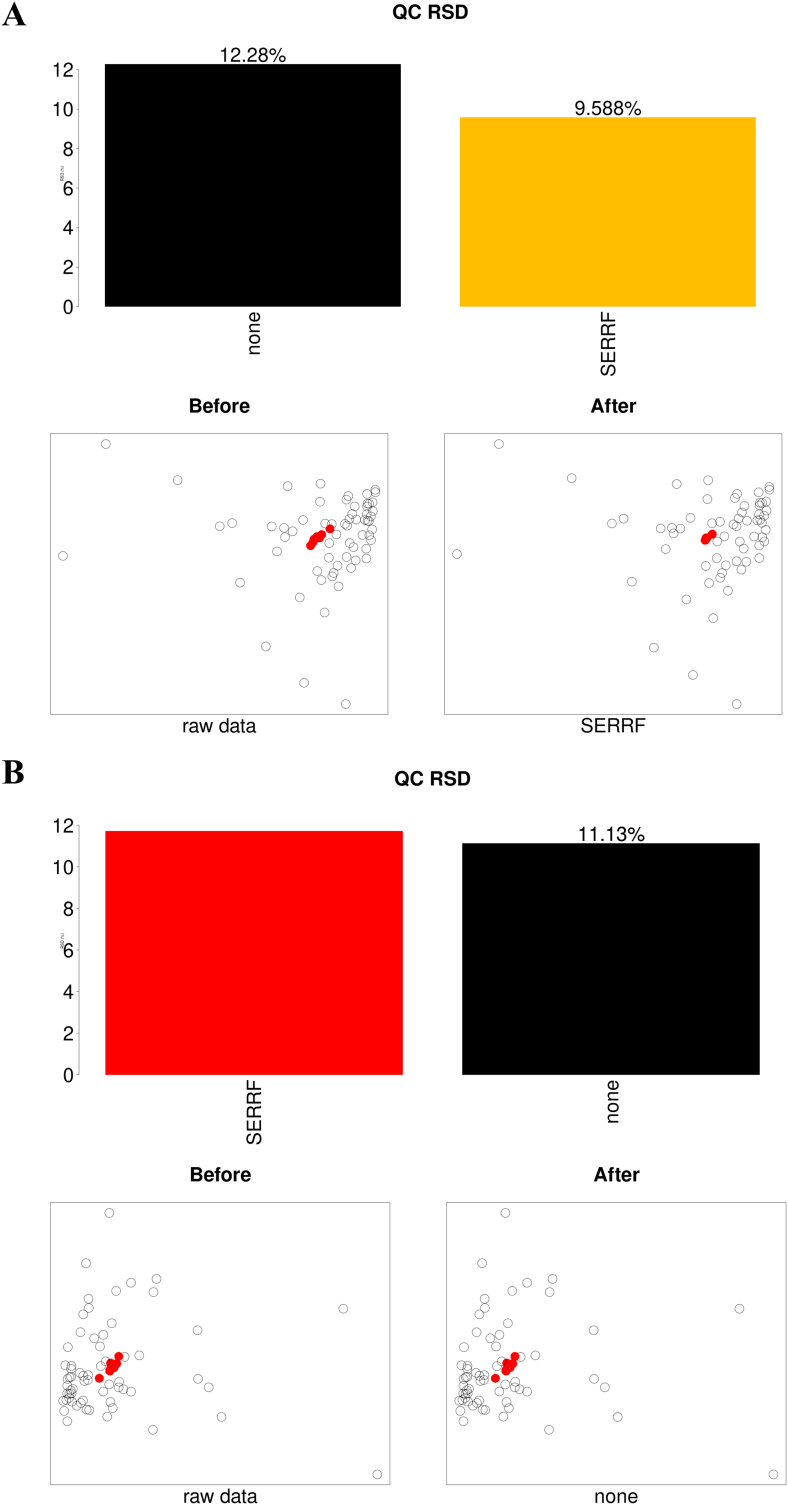
Application of the SERRF normalization method to matrix data from vaginal lavage samples of varying volumes in positive **(A)** and negative **(B)** ion modes. The histograms display the median RSD of peak intensities for all identified metabolites in QC samples. Black bars represent the median RSD of unnormalized data, yellow bars indicate the normalization method with the smallest median RSD, and red bars show the SERRF normalization method, which achieves a slightly smaller median RSD than the yellow bars, indicating its applicability. Below the histograms, PCA score scatter plots illustrate the clustering of QC samples (red circles) and all samples (hollow circles) before and after normalization in both positive and negative ion modes.

The instrument stability and data quality control were accomplished by monitoring the peak height values of internal standards extracted from all raw data generated via LC-MS during sample injection, evaluating the cluster degree of QC samples embedded in the sequence through PCA plots, in addition to calculating the median value of the relative standard deviation (RSD) of all metabolites in QC samples. The critical requirement for acceptance of the stable instrument and successful normalization was the minute variation of QC samples (red circles) in the PCA plots. When data were not normalized, QC samples clustered closely together. Additionally, bar charts above the PCA plots showed the median RSD of all identified metabolites after normalization with the SERRF method. In the positive ion mode, the median RSD decreased from 12.28% pre-normalization to 9.588% post-normalization. In the negative ion mode, the pre-normalization median RSD was 11.13% (**Figures 2A, B**). These results confirmed system stability, and further normalization was unnecessary before downstream analyses.

#### 3.2.2 PCA

**Figure 3:**
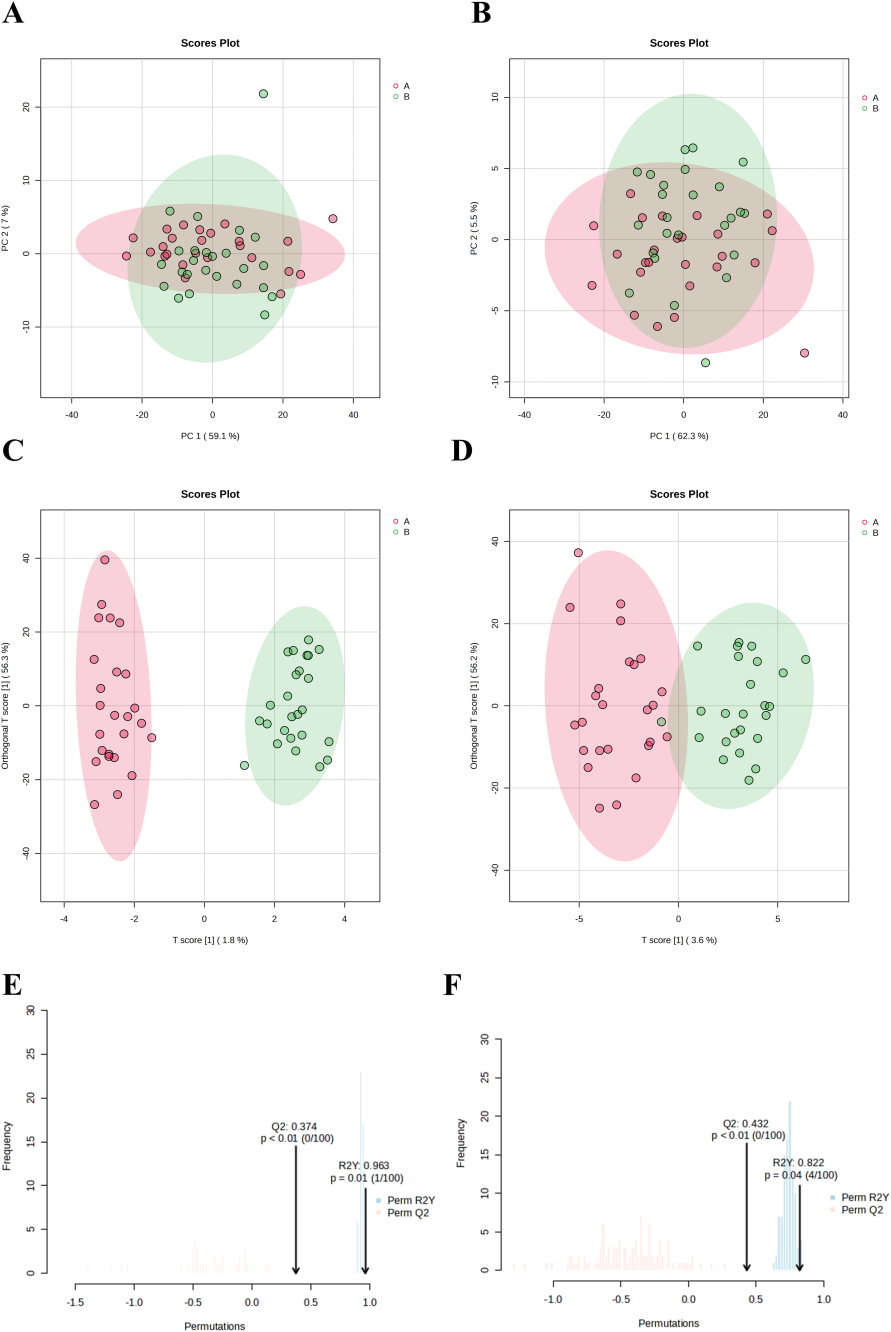
Evaluation and visualization of metabolite clustering in vaginal lavage samples from patients with persistent HPV infection before and after treatment with JWEMG (red region: pre-treatment; green region: post-treatment). **(A)** PCA in positive ion mode for the two groups; **(B)** PCA in negative ion mode for the two groups; **(C)** OPLS-DA in positive ion mode; **(D)** OPLS-DA in negative ion mode. **(E, F)** Permutation tests (n = 100) performed in positive **(E)** and negative **(F)** ion modes to validate the OPLS-DA model. The x-axis represents model accuracy, while the y-axis denotes the frequency of accuracy observed across the 100 permutation tests. The arrow indicates the accuracy of the OPLS-DA model.

PCA was employed to evaluate the differences in vaginal lavage samples from patients with persistent HPV infection before and after treatment with JWEMG. Each point on the PCA score plot represents a sample, with different colors indicating distinct groups. The PCA score plot (**Figures 3A, B**) reveals a separation trend between the two groups, suggesting that significant metabolic changes occurred in the vaginal lavage samples following JWEMG intervention. The evident clustering trend further indicates substantial differences in metabolite expression between the pre- and post-treatment groups.

#### 3.2.3 OPLS-DA

The OPLS-DA method was used to analyze the endogenous metabolites in vaginal lavage samples from patients before and after treatment with JWEMG, in order to identify potential differential metabolites. **Figures 3C, D** show the OPLS-DA score plots under positive and negative ion modes, where the two groups exhibit distinct separation, indicating significant differences between the samples before and after JWEMG treatment. The reliability of the model was validated using permutation testing, with R^2^Y = 0.963 and Q^2^ = 0.374 in the positive ion mode (**Figure 3E**), and R^2^Y = 0.822 and Q^2^ = 0.432 in the negative ion mode (**Figure 3F**). No overfitting was observed, confirming the model’s reliability and allowing further analysis of differential metabolites.

#### 3.2.4 Differential metabolites in vaginal lavage samples before and after treatment

**Figure 4:**
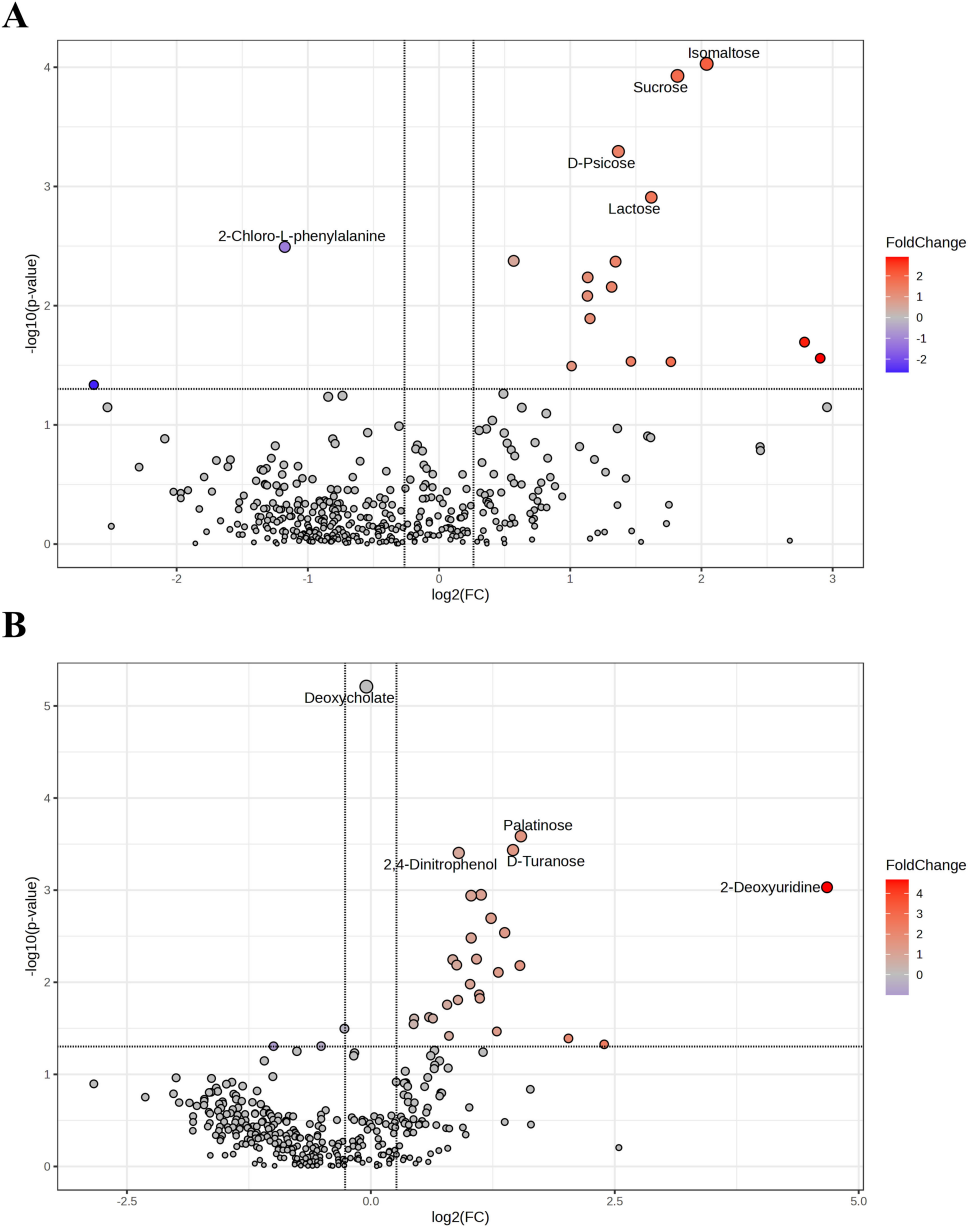
Differential metabolites before and after JWEMG intervention. Volcano plots in the positive ion mode **(A)** and negative ion mode **(B)** illustrate the cumulative differences in metabolites [X-axis: log2(Fold change)] and their statistical significance [Y-axis: -log10(*p*)] in vaginal lavage samples before and after JWEMG intervention. The top five upregulated (red) and downregulated (blue) metabolites after treatment, compared to the pre-treatment samples, are labeled.

Volcano plots were used to visually identify the different metabolites, showing fold changes and statistical significance. Differential metabolites were selected based on a *t*-test with a *p*-value < 0.05 and a Fold change ≥ 1.2 or ≤ 0.8333. In positive ion mode, 17 differential metabolites were identified between the two groups. Compared to pre-treatment, 14 metabolites were upregulated, and 3 were downregulated in the vaginal lavage after treatment. The top 5 differential metabolites are marked in **Figure 4A**. In negative ion mode, a total of 30 differential metabolites were identified, with 16 metabolites upregulated and 14 downregulated in the post-treatment group compared to pre-treatment, as shown in **Figure 4B**. A detailed list of differential metabolites is provided in **Table 3**.

**Table 3:** Differential metabolites between two groups in positive and negative ion modes.

Differential metabolites between two groups in positive ion mode			
Serial number	Metabolite name	*p-*value	Fold change
1	Isomaltose	< 0.001	4.1116
2	Sucrose	< 0.001	3.5238
3	D-Psicose	< 0.001	2.5788
4	Lactose	0.0012336	3.0689
5	2-Chloro-L-phenylalanine	0.0032143	0.4427
6	Dihydrodaidzein	0.0042093	1.4835
7	D-Glucose 6-phosphate	0.0042695	2.5408
8	Maltotetraose	0.005791	2.1933
9	D-(+)-Trehalose	0.0069645	2.4890
10	Maltotriose	0.0082887	2.1901
11	D-Mannose 6-phosphate	0.012834	2.2199
12	3-Methyl-L-histidine	0.020215	6.8952
13	1-Methylhistamine	0.027615	7.4909
14	Putrescine	0.029364	2.7556
15	2’-Deoxyinosine	0.02956	3.4027
16	2’-Deoxyguanosine	0.032124	2.0143
17	Tyr-Arg-Lys	0.046149	0.1614
Differential metabolites between two groups in negative ion mode			
Serial number	Metabolite name	*p-*value	Fold change
1	Palatinose	< 0.001	2.9039
2	D-Turanose	< 0.001	2.7455
3	2,4-Dinitrophenol	< 0.001	1.8672
4	2-Deoxyuridine	< 0.001	25.5560
5	Maltose	0.0011231	2.1884
6	D-Xylose	0.0011473	2.0393
7	Isomaltose	0.0020211	2.3493
8	N-Acetylleucine	0.0028971	2.5879
9	Sebacic acid	0.0033075	2.0397
10	3,4-Dihydroxybenzoic acid	0.005607	2.1185
11	3-Methyl-4-nitrophenol	0.0056826	1.7896
12	Glucose 1-phosphate	0.0065013	1.8406
13	Undecanedioic acid	0.0065899	2.8834
14	Xanthine	0.0078134	2.4738
15	Azelaic acid	0.010485	2.0242
16	D-Fructose 6-phosphate	0.013624	2.1598
17	Maltotriose	0.014967	2.1685
18	Dodecanedioic acid	0.015574	1.8565
19	2’-O-Methylguanosine	0.017545	1.7192
20	N-Isobutyrylglycine	0.02391	1.5131
21	Suberic acid	0.024739	1.5540
22	Benzoic acid	0.024805	1.3600
23	Adipoyl-L-carnitine	0.02853	1.3549
24	L-Glutarylcarnitine	0.031898	0.8289
25	N-Acetyl-L-phenylalanine	0.034204	2.4471
26	Threonic acid	0.038222	1.7418
27	Oxypurinol	0.040737	4.0708
28	2-Isopropylmalic acid	0.047195	5.2477
29	D-Sorbitol	0.049358	0.7023
30	Pro-Leu	0.049458	0.5009

#### 3.2.5 GO enrichment analysis

**Figure 5:**
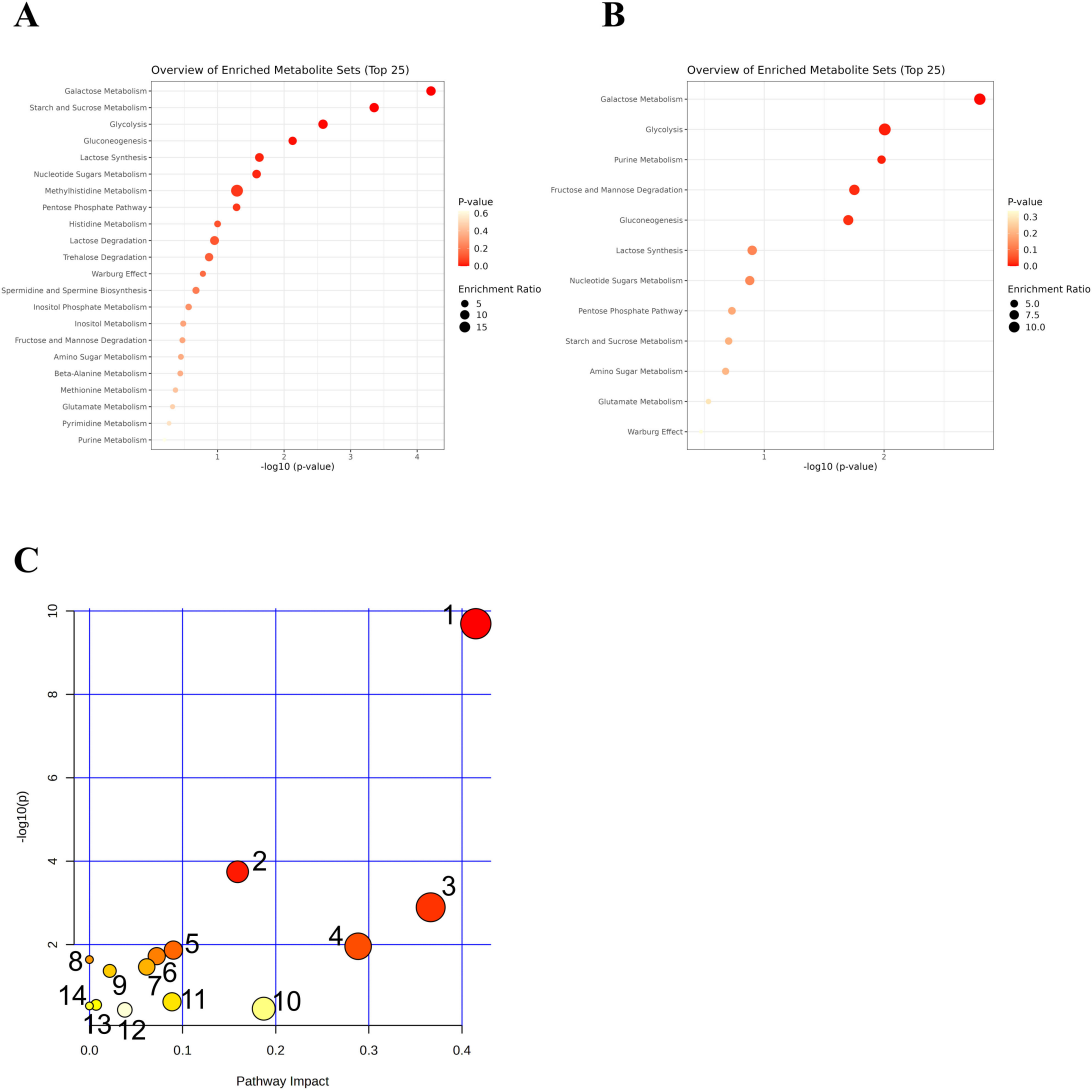
GO enrichment and KEGG pathway analysis of differential metabolites following JWEMG intervention. **(A)** GO terms associated with upregulated metabolites after JWEMG; **(B)** GO terms associated with downregulated metabolites; **(C)** Differential metabolites mapped onto KEGG metabolic pathways. The circle size represents the number of metabolites enriched in each pathway. 1—Starch and sucrose metabolism; 2—Galactose metabolism; 3—Fructose and mannose metabolism; 4—Amino sugar and nucleotide sugar metabolism.

GO functional enrichment analysis was conducted for both upregulated and downregulated metabolites following the treatment with JWEMG, as shown in **Figures 5A, B**. The top 5 enriched terms for the upregulated metabolites included Galactose Metabolism, Starch and Sucrose Metabolism, Glycolysis, Gluconeogenesis, and Lactose Synthesis. For the downregulated metabolites, the top 5 enriched terms were Galactose Metabolism, Glycolysis, Purine Metabolism, Fructose and Mannose Degradation, and Gluconeogenesis.

#### 3.2.6 KEGG pathway analysis

The identified differential metabolites were imported into MetaboAnalyst for pathway analysis, using the hypergeometric test for data analysis. This method aims to identify the metabolic pathways most disturbed after treatment with JWEMG. Pathway impact represents the importance of metabolic pathways, calculated using topological analysis. The −log(*p*) values indicate the statistical significance of the pathway enrichment analysis. Larger pathway impact and −log(*p*) values reflect higher correlations between metabolic differences across groups, with larger circles representing greater relevance. In this study, metabolic pathways with *p* < 0.05 and impact > 0.1 were considered as potential target pathways. A total of four pathways were identified: starch and sucrose metabolism, galactose metabolism, fructose and mannose metabolism, and amino sugar and nucleotide sugar metabolism, as shown in **Figure 5C**. These results suggest that JWEMG may promote HPV clearance by modulating the metabolism of carbohydrates and amino acids in the vaginal/cervical region of patients with persistent HPV infection.

### 3.3 Targeted amino acid metabolomics analysis of vaginal lavage samples in two groups

**Figure 6:**
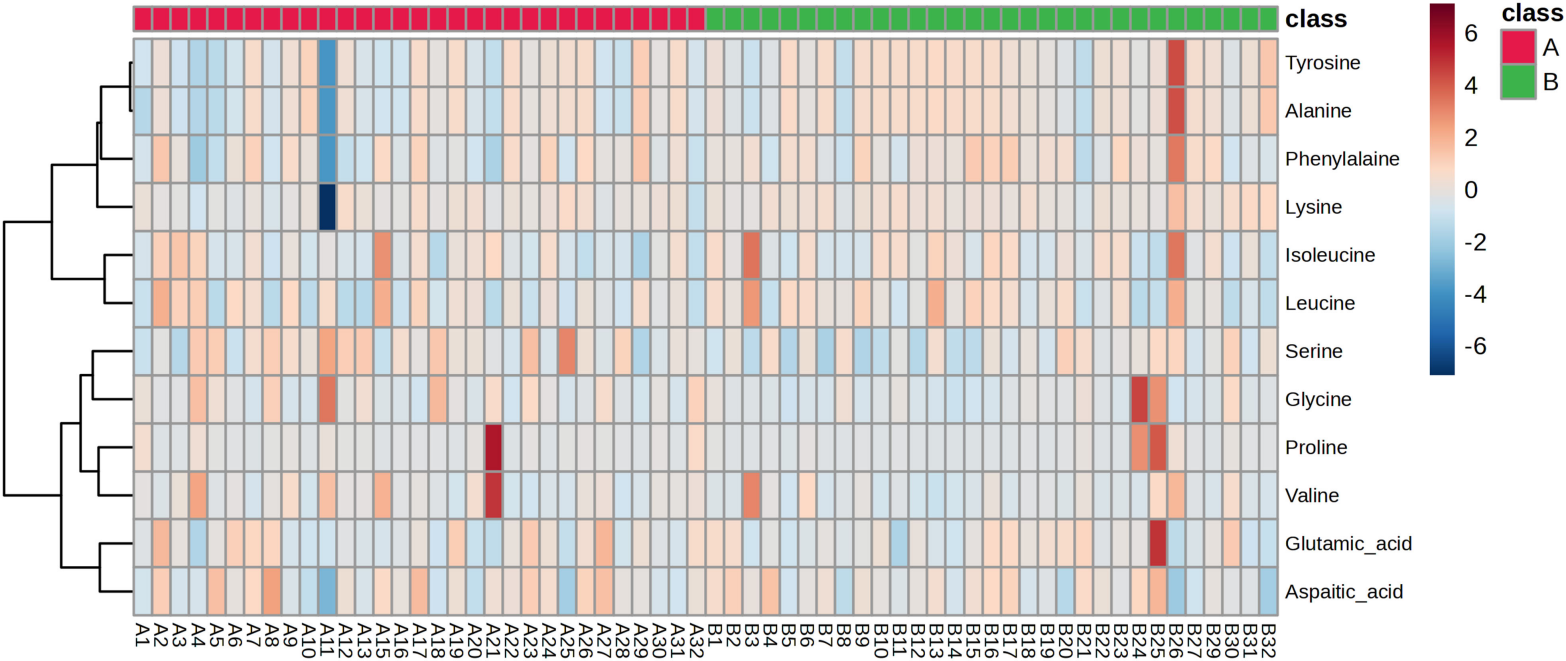
Differential amino acids identified by targeted metabolomics before and after JWEMG treatment.

Building on the above results, we proceeded with targeted amino acid metabolomics analysis of vaginal lavage samples from patients before and after treatment to validate the regulatory effects of JWEMG on amino acid metabolism. During the amino acid quantification process, some amino acids were excluded due to their concentrations being below the instrument’s detection limit or because their signal intensities were too low, leading to unstable quantification. These amino acids were excluded from data analysis to ensure the reliability of the results, including cystine, L-tyrosine, asparagine, histidine, and isoleucine. All amino acid data were imported into MetaboAnalyst software to generate a heatmap (**Figure 6**). Differential amino acids were selected based on a *t*-test with *p* < 0.05 and Fold change ≥ 1.2 or ≤ 0.8333 criteria. The results revealed that tyrosine was the only amino acid with a significant difference between the two groups (*p* = 0.047549). These findings suggest that JWEMG may exert its effects by modulating tyrosine metabolism in the vaginal environment, although the underlying mechanism warrants further investigation.

## 4 Discussion

JWEMG, an in-hospital preparation developed by Jiangsu Provincial Hospital of TCM, has been used for decades to treat cervical HPV infections with a syndrome of damp-heat accumulation syndrome, with well-documented therapeutic efficacy. To further elucidate the pharmacological basis of JWEMG in clearing HR-HPV, our research team employed HPLC in 2018 to quantify active components of JWEMG, including phellodendrine chloride, berberine hydrochloride, jatrorrhizine hydrochloride, (R, S) -goitrine, astilbin, hesperidin and atractylenolideIII. Based on these findings, we established a fingerprint and optimized a stable extraction process (Zou et al., 2018). Further mechanistic studies revealed that berberine, the primary active component of berberine hydrochloride, significantly inhibits the proliferation and migration of cervical cancer HeLa cells by downregulating Bcl-2 expression through the TLR4/NF-κB signaling pathway, thereby promoting apoptosis (Hong et al., 2019). Additionally, JWEMG activates the ALPK1/NF-κB pathway, enhancing the secretion of cytokines and chemokines and restoring local mucosal immunity in the cervix. Metagenomic analysis further demonstrated that JWEMG upregulates the abundance of vaginal *Lactobacillus*, thereby improving the vaginal microenvironment and contributing to the effective clearance of HR-HPV (Li et al., 2025).

In this study, we explored the overall effects of JWEMG on patients with persistent HPV infection through non-targeted metabolomics. We identified 47 differential metabolites, 30 of which showed significant improvement following drug intervention. Our findings suggest that JWEMG may clear HR-HPV infections by regulating pathways such as starch and sucrose metabolism, galactose metabolism, fructose and mannose metabolism, and amino sugar and nucleotide sugar metabolism. To further investigate the impact of JWEMG on amino acid metabolism in the vaginal lavage of HPV patients, we developed a targeted metabolomics approach for vaginal lavage and quantitatively measured 16 amino acids. Using this method, we identified significant differences in the levels of tyrosine in the vaginal lavage before and after treatment, indicating that JWEMG may exert antiviral effects through the modulation of tyrosine metabolism. Our results demonstrate that the combination of targeted and non-targeted metabolomics can play an important role in elucidating the complex mechanisms of TCM. This study provides novel metabolic insights into the multi-component, multi-target mechanisms of JWEMG, further complementing and refining our understanding of its pharmacological actions.

HR-HPV targets various biomolecules, including glucose, amino acids, and lipids, altering their functions (Cruz-Gregorio et al., 2021). HPV can regulate the activity and expression of arginase, which may limit the bioavailability of arginine and inhibit its antiviral properties (Souid et al., 2023). Borgogna JC’s study, using vaginal swab samples for 16S rRNA gene amplicon sequencing, revealed that in women with bacterial vaginosis, HPV-positive patients had significantly lower levels of glutathione-related metabolites, such as oxidized and reduced glutathione, compared to HPV-negative individuals (Borgogna et al., 2020). Other studies that collected vaginal lavage samples, combined with metabolomics, 16S rRNA analysis, and bioinformatics, have elucidated the complex interactions between the virus, host, and microbiota. In patients with LSIL and high-grade squamous intraepithelial lesion (HSIL), vaginal lavage samples exhibited microbiota disruption similar to bacterial vaginosis, affecting amino acid and nucleotide metabolism. Furthermore, several key amino acid metabolites, such as glutamine, pyroglutamine, and N-acetyltaurine, are depleted in HPV-positive or cervical dysplasia participants, distinguishing them from healthy HPV-negative individuals (Ilhan et al., 2019). In 2022, researchers experimentally verified the close relationship between HPV, amino acids, and vaginal microbiota. HPV infection leads to a decrease in antimicrobial peptides secreted by the vaginal/cervical epithelium. Further studies found that antimicrobial peptides, highly expressed by vaginal/cervical keratinocytes, have no antimicrobial activity against *Lactobacillus* but are instead cleaved, internalized, and utilized by *Lactobacillus* to maintain their survival, even when the growth environment of *Lactobacillus* is altered (Lebeau et al., 2022). Therefore, amino acid metabolism plays a crucial role in the occurrence and progression of HPV infection and related diseases.

Tyrosine is a non-essential amino acid that plays a critical role in immune regulation and antiviral mechanisms through tyrosine kinase-mediated signal transduction (Solouki et al., 2019), immune response modulation (Wang et al., 2020), and the immune effects of its metabolites (Loaiza-Cano et al., 2021). The direct relationship between tyrosine metabolism and HPV infection has not yet been clearly established. However, HPV infection can influence host cell signaling and metabolic pathways through the proteins it encodes, including the regulation of tyrosine kinase activity. It has been reported that early proteins E1 (White et al., 2005), E2 (DeSmet et al., 2019; Jose et al., 2020), E5 (Ranieri et al., 2015), E6 (Spangle and Munger, 2013; Lim et al., 2024), and E7 (Bertagnin et al., 2023) interact with tyrosine kinases to affect HPV virus invasion, replication, and carcinogenesis. Additionally, a study collected vaginal swab samples from LSIL, HSIL, and cervical cancer patients for untargeted metabolomics analysis, identifying 155 differential metabolites across the three groups. KEGG pathway enrichment analysis revealed that phenylalanine, tyrosine, and tryptophan biosynthesis pathways play a key role in the development of cervical cancer (Xu et al., 2022). In the present study, although not all amino acid standards were tested, we found that tyrosine was the only significantly altered amino acid following JWEMG treatment. Therefore, future research will focus on exploring the metabolic pathways of tyrosine and its potential association with the clearance of HPV by JWEMG, further validating the specific metabolic changes in tyrosine.

Certainly, there are some limitations to our study. This was a single-center, small-sample, prospective study, with all samples collected from one medical center. The potential biomarkers identified through microbiome and metabolomic analyses need further validation in larger clinical cohorts. Additionally, future causal relationship studies will be necessary to better understand the disease mechanisms. Furthermore, although all patients had cervical lesions classified as LSIL or lower, studies have shown that there are metabolic differences even between LSIL and HPV-negative patients (Yu et al., 2024), indicating the need for more detailed stratification of cervical lesions. Another limitation is the small sample size of the non-cleared infection group, which precluded a robust metabolomic comparison between patients who cleared the infection and those who did not. Such an analysis could have provided valuable insights into the metabolic patterns associated with JWEMG’s clinical efficacy in non-responders. Finally, the role of vaginal microbiota in HPV clearance has been extensively studied. Our team has analyzed the vaginal lavage samples of these patients through metagenomics, and the results have recently been published. In the future, we plan to conduct an integrated analysis of metagenomics and metabolomics data to explore the interaction between these two factors and further uncover the potential mechanisms underlying the HPV clearance effects of JWEMG.

## 5 Conclusion

In this study, we established a non-targeted metabolomics analysis approach to explore the potential mechanisms of JWEMG in treating patients with persistent HPV infection. The results revealed 47 differential metabolites identified in vaginal lavage samples before and after treatment. Pathway analysis indicated that JWEMG may exert antiviral effects through metabolic pathways such as starch and sucrose metabolism, galactose metabolism, fructose and mannose metabolism, and amino sugar and nucleotide sugar metabolism. Subsequently, we conducted a targeted metabolomics study to quantitatively analyze 16 amino acids and found that JWEMG may clear HPV by targeting tyrosine metabolism. Our study not only investigates the potential therapeutic effects of JWEMG in clearing persistent HPV infection but also provides valuable insights into elucidating the complex mechanisms of traditional Chinese medicine.

## Data Availability

The original contributions presented in the study are included in the article/supplementary material. Further inquiries can be directed to the corresponding author.
